# Supplementary material for: Underlying mechanisms of oxygen uptake kinetics in chronic post-stroke individuals: A correlational, cross-sectional pilot study
Source: PLoS One. 2020 Nov 9;15(11):e0241872. doi: 10.1371/journal.pone.0241872 (PMC7652273; doi:10.1371/journal.pone.0241872)
Supplement: S1 File — (DOCX) [file pone.0241872.s003.docx]

**Syntax file**

NONPAR CORR

/MISSING = LISTWISE

/MATRIX OUT(*).

RECODE rowtype_ ('RHO'='CORR') .

PARTIAL CORR

/significance = twotail

/MISSING = LISTWISE

/MATRIX IN(*).

**S1 Table** Relative oxygen uptake during the six-minute walk test for each individual.

| **Participant** | **V̇O_2_ (mL/kg/min)** | | | | | |
| --- | --- | --- | --- | --- | --- | --- |
|  | **1º minute** | **2º minute** | **3º minute** | **4º minute** | **5º minute** | **6º minute** |
| 01AJC | 4.48 (1.79) | 6.95 (1.55) | 7.93 (0.91) | 7.77 (0.89) | 7.73 (0.79) | 7.90 (1.14) |
| 02WP | 6.81 (1.89) | 10.31 (0.98) | 10.82 (1.62) | 10.37 (1.47) | 10.55 (1.05) | 9.86 (1.39) |
| 03AMB | 8.33 (2.34) | 10.23 (1.88) | 10.96 (1.22) | 11.07 (1.63) | 10.47 (1.68) | 10.82 (1.79) |
| 04EAP | 5.71 (1.10) | 7.38 (1.08) | 7.39 (1.61) | 7.43 (1.23) | 7.40 (0.80) | 7.39 (1.11) |
| 05PPS | 10.33 (3.99) | 17.55 (1.89) | 16.90 (1.93) | 17.00 (1.64) | 16.97 (1.68) | 17.20 (1.86) |
| 06ACL | 7.17 (1.28) | 9.42 (1.15) | 9.13 (0.95) | 8.84 (0.58) | 8.88 (0.59) | 8.51 (1.54) |
| 07AAO | 6.35 (0.97) | 8.27 (1.19) | 8.87 (0.96) | 7.68 (0.85) | 7.92 (0.73) | 7.35 (1.17) |
| 08GAJ | 5.63 (1.08) | 7.12 (0.81) | 7.63 (0.71) | 7.68 (0.74) | 7.51 (0.63) | 7.50 (0.81) |
| 09SPM | 7.61 (1.71) | 10.26 (1.92) | 10.44 (1.11) | 10.66 (1.33) | 10.47 (1.46) | 11.12 (0.90) |
| 10LTJ | 5.16 (0.82) | 7.41 (1.20) | 7.95 (1.06) | 8.58 (0.64) | 8.06 (1.29) | 8.80 (0.84) |
| 11OAS | 8.31 (1.72) | 11.64 (0.72) | 11.27 (0.72) | 11.08 (0.59) | 10.80 (0.84) | 10.97 (0.52) |
| 12JRS | 8.61 (1.89) | 12.68 (1.43) | 14.41 (1.80) | 14.22 (1.44) | 13.80 (1.39) | 14.06 (1.62) |
| 13JA | 8.62 (2.83) | 11.59 (2.01) | 12.18 (2.06) | 12.30 (1.29) | 12.22 (1.14) | 12.04 (0.95) |
| 14EAB | 7.12 (1.41) | 10.20 (0.98) | 10.88 (0.63) | 10.82 (0.79) | 10.92 (0.70) | 10.95 (0.69) |
| 15VRS | 8.80 (1.81) | 11.94 (0.66) | 12.52 (0.44) | 12.30 (0.42) | 12.26 (0.45) | 12.55 (0.46) |
| 16SRB | 8.80 (2.46) | 12.49 (1.93) | 13.39 (1.24) | 13.59 (1.59) | 13.94 (1.28) | 13.94 (1.32) |
| 17MLG | 7.94 (1.78) | 9.72 (1.86) | 10.33 (1.69) | 9.87 (1.00) | 10.74 (1.78) | 10.03 (1.59) |
| 18ABC | 5.36 (1.02) | 7.42 (0.73) | 8.18 (0.66) | 8.18 (0.76) | 8.28 (0.61) | 8.16 (0.48) |
| 19FLN | 8.57 (1.32) | 10.99 (0.95) | 11.05 (1.08) | 10.93 (0.83) | 10.95 (0.90) | 11.32 (1.20) |
| 20FFA | 8.01 (1.65) | 11.10 (1.32) | 11.50 (0.89) | 11.81 (0.71) | 11.76 (1.22) | 11.71 (1.11) |
| 21ASO | 7.96 (1.46) | 9.96 (1.65) | 10.40 (1.21) | 10.28 (1.79) | 10.36 (1.21) | 10.51 (1.71) |
| 22MGS | 10.01 (3.04) | 13.41 (2.40) | 14.18 (1.86) | 14.50 (1.66) | 14.60 (1.70) | 14.93 (1.86) |
| 23PLS | 8.85 (1.71) | 11.90 (1.16) | 12.68 (0.61) | 12.29 (0.73) | 12.14 (0.50) | 11.97 (0.39) |
| 24JAF | 8.62 (1.45) | 10.20 (1.32) | 10.76 (0.94) | 10.40 (0.87) | 10.42 (1.01) | 10.53 (1.21) |

**Note:** Oxygen uptake is reported as mean (standard deviation). Note that all participants had standard deviation of relative oxygen uptake over the last three minutes ≤ 2.0 mL/kg/min (steady-state condition).

**Abbreviations:** mL/kg/min, milliliter per kilogram per minute; V̇O_2_, oxygen uptake.

**S2 Table** Respiratory exchange ratio during the six-minute walk test for each individual.

| **Participant** | **RER** | | | | | |
| --- | --- | --- | --- | --- | --- | --- |
|  | **1º minute** | **2º minute** | **3º minute** | **4º minute** | **5º minute** | **6º minute** |
| 01AJC | 0.99 (0.06) | 0.86 (0.02) | 0.91 (0.03) | 0.96 (0.02) | 0.98 (0.02) | 1.00 (0.05) |
| 02WP | 0.87 (0.05) | 0.87 (0.02) | 0.93 (0.03) | 0.94 (0.04) | 0.96 (0.02) | 0.97 (0.03) |
| 03AMB | 0.85 (0.06) | 0.87 (0.04) | 0.90 (0.04) | 0.94 (0.04) | 0.94 (0.03) | 0.93 (0.04) |
| 04EAP | 0.99 (0.04) | 0.93 (0.03) | 0.90 (0.03) | 0.92 (0.04) | 0.96 (0.03) | 0.97 (0.04) |
| 05PPS | 0.97 (0.11) | 0.99 (0.06) | 1.07 (0.04) | 1.06 (0.02) | 1.05 (0.03) | 1.06 (0.02) |
| 06ACL | 1.02 (0.04) | 1.00 (0.03) | 1.03 (0.02) | 1.04 (0.02) | 1.04 (0.02) | 1.03 (0.04) |
| 07AAO | 0.91 (0.03) | 0.85 (0.02) | 0.91 (0.02) | 0.91 (0.01) | 0.91 (0.02) | 0.90 (0.04) |
| 08GAJ | 0.88 (0.05) | 0.82 (0.02) | 0.90 (0.03) | 0.95 (0.02) | 1.01 (0.02) | 1.01 (0.02) |
| 09SPM | 0.83 (0.03) | 0.81 (0.03) | 0.87 (0.02) | 0.90 (0.03) | 0.92 (0.03) | 0.94 (0.03) |
| 10LTJ | 0.91 (0.04) | 0.88 (0.01) | 0.90 (0.03) | 0.96 (0.02) | 0.95 (0.01) | 0.99 (0.02) |
| 11OAS | 0.97 (0.08) | 0.92 (0.03) | 0.98 (0.01) | 1.00 (0.01) | 1.00 (0.02) | 1.03 (0.01) |
| 12JRS | 0.90 (0.03) | 0.87 (0.02) | 0.91 (0.03) | 0.95 (0.02) | 0.97 (0.01) | 1.00 (0.01) |
| 13JA | 1.26 (1.42) | 0.83 (0.05) | 0.86 (0.05) | 0.91 (0.04) | 0.95 (0.04) | 1.01 (0.03) |
| 14EAB | 0.85 (0.02) | 0.87 (0.03) | 0.93 (0.01) | 0.93 (0.02) | 0.93 (0.01) | 0.96 (0.02) |
| 15VRS | 1.00 (0.04) | 1.09 (0.02) | 1.11 (0.02) | 1.09 (0.01) | 1.08 (0.01) | 1.07 (0.01) |
| 16SRB | 0.88 (0.06) | 0.86 (0.03) | 0.93 (0.03) | 0.96 (0.05) | 0.99 (0.01) | 1.01 (0.02) |
| 17MLG | 0.92 (0.05) | 0.87 (0.06) | 0.92 (0.04) | 0.97 (0.02) | 0.92 (0.06) | 0.96 (0.04) |
| 18ABC | 0.73 (0.02) | 0.68 (0.02) | 0.73 (0.02) | 0.75 (0.01) | 0.76 (0.02) | 0.77 (0.02) |
| 19FLN | 0.78 (0.03) | 0.76 (0.02) | 0.79 (0.02) | 0.79 (0.01) | 0.80 (0.01) | 0.80 (0.02) |
| 20FFA | 0.79 (0.04) | 0.79 (0.03) | 0.82 (0.02) | 0.84 (0.02) | 0.85 (0.04) | 0.84 (0.03) |
| 21ASO | 0.85 (0.02) | 0.81 (0.03) | 0.82 (0.02) | 0.83 (0.01) | 0.84 (0.02) | 0.86 (0.03) |
| 22MGS | 0.80 (0.06) | 0.83 (0.03) | 0.89 (0.03) | 0.90 (0.01) | 0.92 (0.02) | 0.92 (0.03) |
| 23PLS | 0.75 (0.06) | 0.73 (0.03) | 0.77 (0.02) | 0.78 (0.01) | 0.80 (0.01) | 0.81 (0.01) |
| 24JAF | 0.79 (0.04) | 0.80 (0.03) | 0.83 (0.03) | 0.85 (0.02) | 0.87 (0.03) | 0.86 (0.02) |

**Note:** Respiratory exchange ratio is reported as mean (standard deviation). Note that all participants had respiratory exchange ratio values over the last three minutes < 1.1 (steady-state condition).

**Abbreviations:** RER, respiratory exchange ratio.

**S3 Table** Metabolic response during the six-minute walk test for each individual.

| **Participant** | **V̇O_2_ (mL/kg/min)** | | | | | | **p** |
| --- | --- | --- | --- | --- | --- | --- | --- |
|  | **1º minute** | **2º minute** | **3º minute** | **4º minute** | **5º minute** | **6º minute** |  |
| 01AJC | 4.85 (2.88 to 5.70) | 7.30 (6.50 to 7.50) | 8.00 (7.33 to 8.58) | 8.05 (7.18 to 8.40) | 7.80 (7.25 to 8.28) | 7.80 (7.15 to 8.60) | 0.800 |
| 02WP | 7.10 (5.40 to 8.60) | 10.30 (9.70 to 11.10) | 10.80 (9.90 to 11.70) | 10.70 (9.50 to 11.10) | 10.50 (9.85 to 11.30) | 9.90 (8.98 to 11.05) | 0.198 |
| 03AMB | 8.90 (5.93 to 10.40) | 10.65 (8.42 to 11.45) | 10.75 (10.13 to 11.75) | 11.55 (9.90 to 12.13) | 11.00 (8.80 to 11.80) | 10.90 (9.40 to 12.10) | 0.486 |
| 04EAP | 5.80 (5.20 to 6.45) | 7.20 (6.80 to 8.13) | 7.65 (6.40 to 8.88) | 7.40 (6.70 to 8.40) | 7.35 (6.90 to 7.90) | 7.60 (7.20 to 8.05) | 0.872 |
| 05PPS | 11.80 (6.23 to 13.25) | 18.10 (16.40 to 18.85) | 17.20 (15.75 to 18.40) | 17.15 (16.05 to 17.58) | 16.90 (15.70 to 18.40) | 17.00 (15.60 to 19.08) | 0.667 |
| 06ACL | 7.30 (5.90 to 8.50) | 9.20 (8.83 to 10.00) | 9.15 (8.80 to 9.58) | 8.90 (8.50 to 9.20) | 8.80 (8.35 to 9.40) | 8.35 (7.40 to 9.35) | 0.304 |
| 07AAO | 6.20 (5.90 to 6.68) | 8.40 (7.60 to 9.00) | 8.75 (8.17 to 9.15) | 7.80 (7.10 to 8.30) | 8.10 (7.53 to 8.40) | 7.60 (6.80 to 8.10) | 0.319 |
| 08GAJ | 5.55 (4.85 to 6.35) | 7.30 (6.50 to 7.70) | 7.80 (7.15 to 8.05) | 7.80 (7.10 to 8.35) | 7.45 (7.05 to 7.98) | 7.70 (6.90 to 8.00) | 0.924 |
| 09SPM | 8.25 (5.80 to 8.93) | 10.40 (8.88 to 11.93) | 10.55 (9.33 to 11.40) | 10.85 (10.08 to 11.40) | 10.85 (9.93 to 11.20) | 10.90 (10.50 to 11.75) | 0.745 |
| 10LTJ | 5.05 (4.65 to 5.63) | 7.10 (6.55 to 8.40) | 7.90 (7.43 to 8.65) | 8.70 (8.05 to 9.05) | 8.35 (7.33 to 8.75) | 9.00 (8.00 to 9.50) | 0.186 |
| 11OAS | 7.80 (7.23 to 10.00) | 11.80 (11.15 to 12.30) | 11.40 (10.85 to 11.75) | 11.10 (10.70 to 11.55) | 10.75 (10.30 to 11.10) | 11.00 (10.63 to 11.48) | 0.484 |
| 12JRS | 8.80 (6.80 to 10.00) | 12.75 (11.78 to 13.83) | 14.40 (13.35 to 15.50) | 14.50 (12.90 to 15.20) | 13.70 (13.00 to 14.60) | 14.40 (12.70 to 15.40) | 0.776 |
| 13JA | 8.25 (6.55 to 10.48) | 11.35 (10.13 to 13.08) | 11.60 (10.50 to 14.20) | 12.30 (11.48 to 13.10) | 12.25 (11.40 to 13.15) | 12.15 (11.60 to 12.68) | 0.843 |
| 14EAB | 6.95 (6.05 to 8.45) | 10.30 (9.75 to 10.85) | 10.90 (10.40 to 11.40) | 10.80 (10.40 to 11.40) | 10.90 (10.75 to 11.30) | 11.05 (10.67 to 11.33) | 0.729 |
| 15VRS | 9.25 (7.18 to 10.05) | 12.10 (11.60 to 12.30) | 12.50 (12.28 to 12.77) | 12.20 (12.00 to 12.60) | 12.30 (11.85 to 12.55) | 12.50 (12.30 to 12.90) | 0.065 |
| 16SRB | 8.30 (7.70 to 9.50) | 12.40 (11.30 to 13.80) | 13.60 (12.50 to 14.10) | 13.70 (12.33 to 15.08) | 13.95 (13.20 to 15.30) | 14.10 (12.90 to 14.90) | 0.675 |
| 17MLG | 8.10 (6.30 to 9.20) | 9.65 (8.58 to 10.38) | 10.70 (8.40 to 11.55) | 9.90 (8.95 to 10.60) | 10.90 (9.10 to 12.50) | 10.90 (9.10 to 12.50) | 0.774 |
| 18ABC | 5.50 (4.73 to 5.95) | 7.40 (6.80 to 7.90) | 8.20 (7.88 to 8.60) | 8.20 (7.70 to 8.60) | 8.30 (7.83 to 8.70) | 8.20 (7.75 to 8.50) | 0.856 |
| 19FLN | 8.70 (7.65 to 9.55) | 11.10 (10.80 to 11.50) | 11.15 (10.58 to 11.77) | 10.90 (10.30 to 11.60) | 11.10 (10.40 to 11.45) | 11.60 (10.50 to 12.10) | 0.368 |
| 20FFA | 8.00 (7.30 to 9.10) | 11.20 (10.43 to 12.15) | 11.55 (10.70 to 12.20) | 11.95 (11.23 to 12.33) | 12.10 (11.10 to 12.63) | 11.70 (11.17 to 12.50) | 0.544 |
| 21ASO | 8.20 (6.65 to 9.05) | 10.30 (9.60 to 10.67) | 10.40 (9.65 to 11.15) | 10.25 (9.03 to 11.25) | 10.70 (9.60 to 11.10) | 10.60 (9.50 to 12.00) | 0.926 |
| 22MGS | 9.60 (7.58 to 12.88) | 13.05 (12.00 to 14.85) | 14.15 (12.70 to 15.93) | 14.65 (13.93 to 15.60) | 14.90 (13.45 to 15.85) | 14.70 (13.50 to 16.70) | 0.483 |
| 23PLS | 8.60 (8.10 to 9.90) | 11.90 (11.20 to 12.80) | 12.80 (12.35 to 13.05) | 12.30 (12.00 to 12.85) | 12.20 (11.95 to 12.40) | 11.90 (11.60 to 12.40) | 0.113 |
| 24JAF | 8.90 (7.70 to 9.55) | 10.45 (9.83 to 10.88) | 11.10 (9.90 to 11.50) | 10.40 (9.75 to 11.20) | 10.55 (9.30 to 11.13) | 10.65 (9.75 to 11.40) | 0.479 |

**Note:** Oxygen uptake is reported as median (interquartile range). We used the Friedman’s ANOVA test to determine if there was a significant difference of relative oxygen uptake among the last three minutes of the six-minute walk test. Note that all participants had no difference in relative oxygen uptake among the last three minutes (steady-state condition).

**Abbreviations:** mL/kg/min, milliliter per kilogram per minute; V̇O_2_, oxygen uptake.

*p ≤ 0.05

**S4 Table** Relationship between the oxygen uptake kinetics and the underlying mechanisms in the adult group (19-59 years, n = 9).

| **Variables** | **wMRT_ON_ (min^2^/mL/kg)** | | **wMRT_OFF_ (min^2^/mL/kg)** | |
| --- | --- | --- | --- | --- |
|  | **r_s_ [BCa CI_95_]** | **p** | **r_s_ [BCa CI_95_]** | **p** |
| Body Composition |  |  |  |  |
| Weight (kg) | 0.42 [-0.25, 0.94] | 0.26 | 0.44 [-0.29, 0.88] | 0.24 |
| Body mass index (kg/m^2^) | 0.32 [-0.46, 0.87] | 0.40 | 0.36 [-0.39, 0.74] | 0.34 |
| Body fat mass (kg) | 0.33 [-0.41, 0.87] | 0.39 | 0.39 [-0.31, 0.82] | 0.29 |
| Body fat mass index (kg/m^2^) | 0.38 [-0.56, 0.98] | 0.31 | 0.47 [-0.32, 0.90] | 0.20 |
| Skeletal muscle mass (kg) | 0.23 [-0.67, 0.92] | 0.55 | 0.15 [-0.46, 0.58] | 0.70 |
| Skeletal muscle mass index (kg/m^2^) | 0.15 [-0.72, 0.92] | 0.70 | 0.20 [-0.45, 0.62] | 0.61 |
| Arterial compliance |  |  |  |  |
| cfPWV^a^ (m/s) | -0.61 [-0.95, 0.10] | 0.11 | -0.35 [-0.97, 0.54] | 0.40 |
| AIx (%) | 0.18 [-0.72, 0.87] | 0.64 | 0.25 [-0.75, 0.84] | 0.52 |
| AIx75 (%) | 0.39 [-0.30, 0.86] | 0.29 | 0.50 [-0.37, 0.95] | 0.17 |
| Reflection magnitude (%) | 0.22 [-0.68, 0.90] | 0.58 | 0.23 [-0.78, 0.84] | 0.55 |
| Endothelial Function |  |  |  |  |
| baFMD (%) | -0.23 [-0.72, 0.35] | 0.56 | -0.56 [-1.00, 0.35] | 0.11 |
| Hematological and inflammatory profiles |  |  |  |  |
| Erythrocyte (million/mm^3^) | -0.13 [-0.72, 0.63] | 0.74 | -0.35 [-0.95, 0.66] | 0.35 |
| Hemoglobin (g/dL) | -0.05 [-0.70, 0.70] | 0.90 | -0.17 [-0.87, 0.82] | 0.66 |
| Hematocrit (%) | -0.08 [-0.69, 0.59] | 0.85 | -0.25 [-0.90, 0.61] | 0.51 |
| High-sensitivity C-reactive protein (mg/L) | 0.59 [-0.18, 1.00] | 0.09 | 0.56 [-0.27, 0.95] | 0.11 |

**Note:** 95% bias corrected and accelerated confidence intervals reported in square brackets. Confidence intervals based on 10,000 bootstrap samples.

**Abbreviations:** %, percentage; AIx, augmentation index; AIx75, augmentation index normalized to a heart rate of 75 bpm; baFMD, brachial artery flow-mediated dilation; BCa, bias corrected accelerated; cfPWV, carotid-femoral pulse wave velocity; CI_95_, 95% confidence interval; g/dL, grams per deciliter; kg, kilogram; kg/m^2^, kilogram per meter^2^; m/s, meter per second; mg/L, milligram per liter; million/mm^3^, million per cubic millimeter; min^2^/mL/kg, minute square per milliliter per kilogram; r_s_, Spearman’s rank correlation coefficient; wMRT_OFF_, oxygen uptake off-kinetics mean response time corrected for work rate; wMRT_ON_, oxygen uptake on-kinetics mean response time corrected for work rate.

*p ≤ 0.05

^a^n = 8

**S5 Table** Relationship between the oxygen uptake kinetics and the underlying mechanisms in the elderly group (≥ 60 years, n = 15).

| **Variables** | **wMRT_ON_ (min^2^/mL/kg)** | | **wMRT_OFF_ (min^2^/mL/kg)** | |
| --- | --- | --- | --- | --- |
|  | **r_s_ [BCa CI_95_]** | **p** | **r_s_ [BCa CI_95_]** | **p** |
| Body Composition |  |  |  |  |
| Weight (kg) | -0.01 [-0.59, 0.55] | 0.98 | -0.59 [-0.90, 0.00] | 0.02* |
| Body mass index (kg/m^2^) | 0.02 [-0.49, 0.50] | 0.94 | -0.49 [-0.86, 0.13] | 0.07 |
| Body fat mass^c^ (kg) | -0.07 [-0.53, 0.38] | 0.82 | -0.45 [-0.79, 0.02] | 0.11 |
| Body fat mass index^c^ (kg/m^2^) | -0.12 [-0.57, 0.40] | 0.69 | -0.34 [-0.70, 0.12] | 0.24 |
| Skeletal muscle mass^c^ (kg) | -0.15 [-0.61, 0.41] | 0.61 | -0.49 [-0.83, 0.10] | 0.08 |
| Skeletal muscle mass index^c^ (kg/m^2^) | -0.16 [-0.64, 0.41] | 0.58 | -0.71 [-0.97, -0.22] | < 0.01* |
| Arterial compliance |  |  |  |  |
| cfPWV^a^ (m/s) | 0.23 [-0.47, 0.77] | 0.50 | 0.17 [-0.58, 0.75] | 0.63 |
| AIx^c^ (%) | -0.18 [-0.71, 0.41] | 0.55 | 0.23 [-0.35, 0.72] | 0.42 |
| AIx75^c^ (%) | 0.08 [-0.56, 0.66] | 0.78 | 0.49 [-0.18, 0.94] | 0.07 |
| Reflection magnitude^c^ (%) | -0.05 [-0.62, 0.58] | 0.86 | 0.30 [-0.33, 0.79] | 0.29 |
| Endothelial Function^b^ |  |  |  |  |
| baFMD (%) | -0.47 [-0.86, 0.07] | 0.11 | -0.24 [-0.71, 0.27] | 0.43 |
| Hematological and inflammatory profiles^b^ |  |  |  |  |
| Erythrocyte (million/mm^3^) | -0.08 [-0.67, 0.56] | 0.79 | -0.43 [-0.80, 0.14] | 0.14 |
| Hemoglobin (g/dL) | 0.01 [-0.60, 0.71] | 0.98 | -0.33 [-0.77, 0.40] | 0.27 |
| Hematocrit (%) | 0.08 [-0.50, 0.62] | 0.79 | -0.34 [-0.80, 0.37] | 0.26 |
| High-sensitivity C-reactive protein (mg/L) | 0.01 [-0.50, 0.50] | 0.98 | 0.48 [-0.02, 0.81] | 0.09 |

**Note:** 95% bias corrected and accelerated confidence intervals reported in square brackets. Confidence intervals based on 10,000 bootstrap samples.

**Abbreviations:** %, percentage; AIx, augmentation index; AIx75, augmentation index normalized to a heart rate of 75 bpm; baFMD, brachial artery flow-mediated dilation;

BCa, bias corrected accelerated; cfPWV, carotid-femoral pulse wave velocity; CI_95_, 95% confidence interval; g/dL, grams per deciliter; kg, kilogram; kg/m^2^, kilogram per meter^2^; m/s, meter per second; mg/L, milligram per liter; million/mm^3^, million per cubic millimeter; min^2^/mL/kg, minute square per milliliter per kilogram; r_s_, Spearman’s rank correlation coefficient; wMRT_OFF_, oxygen uptake off-kinetics mean response time corrected for work rate; wMRT_ON_, oxygen uptake on-kinetics mean response time corrected for work rate.

*p ≤ 0.05; ^a^n = 11

^b^n = 13

^c^n = 14

**
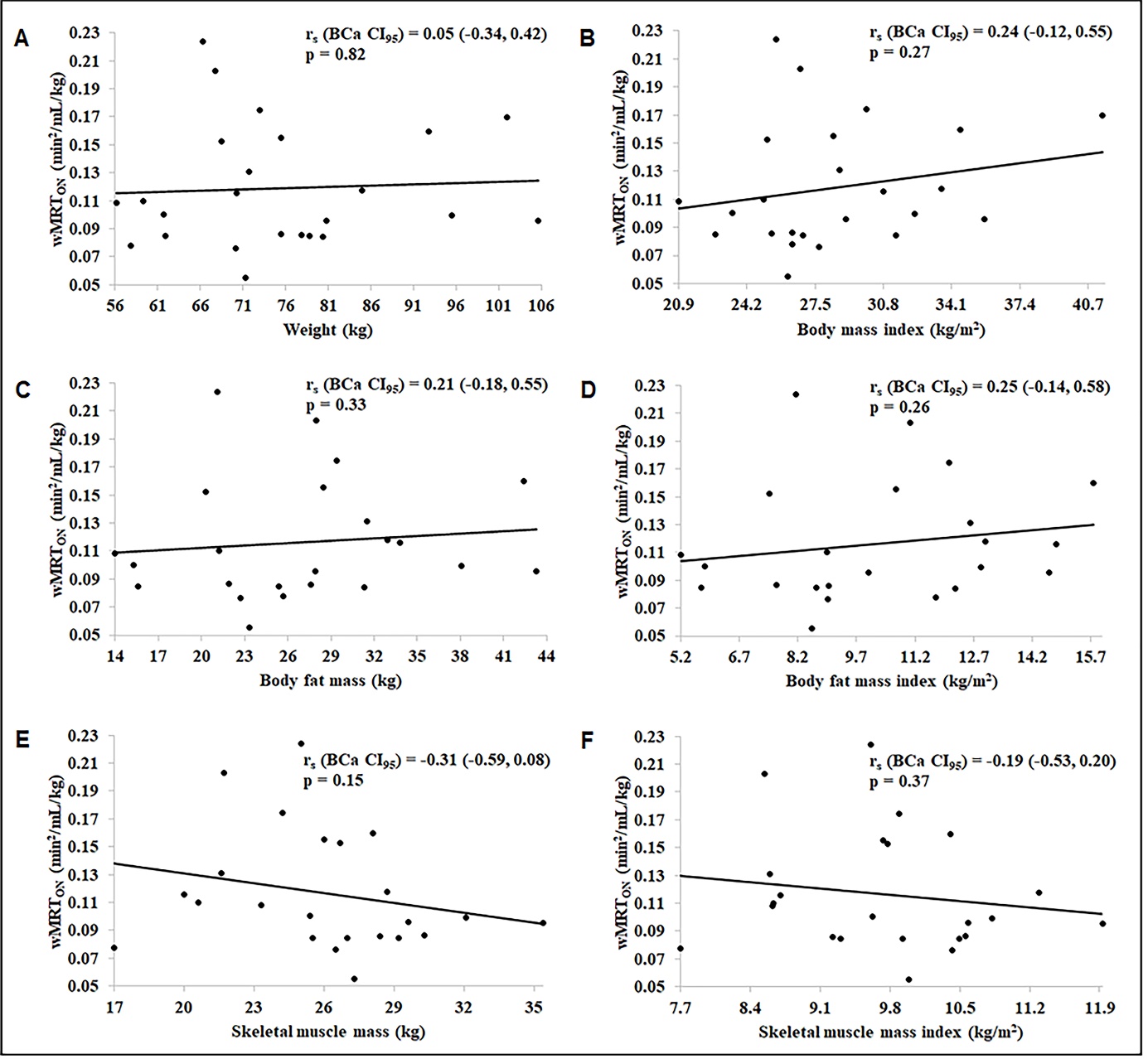
**

**S1 Fig** Relationship between the body composition and the oxygen uptake on-kinetics mean response time corrected for work rate. BCa, bias corrected accelerated; CI_95_, 95% confidence interval; kg, kilogram; kg/m^2^, kilogram per meter^2^; min^2^/mL/kg, minute square per milliliter per kilogram; r_s_, Spearman’s rank correlation coefficient; wMRT_ON_, oxygen uptake on-kinetics mean response time corrected for work rate.

**
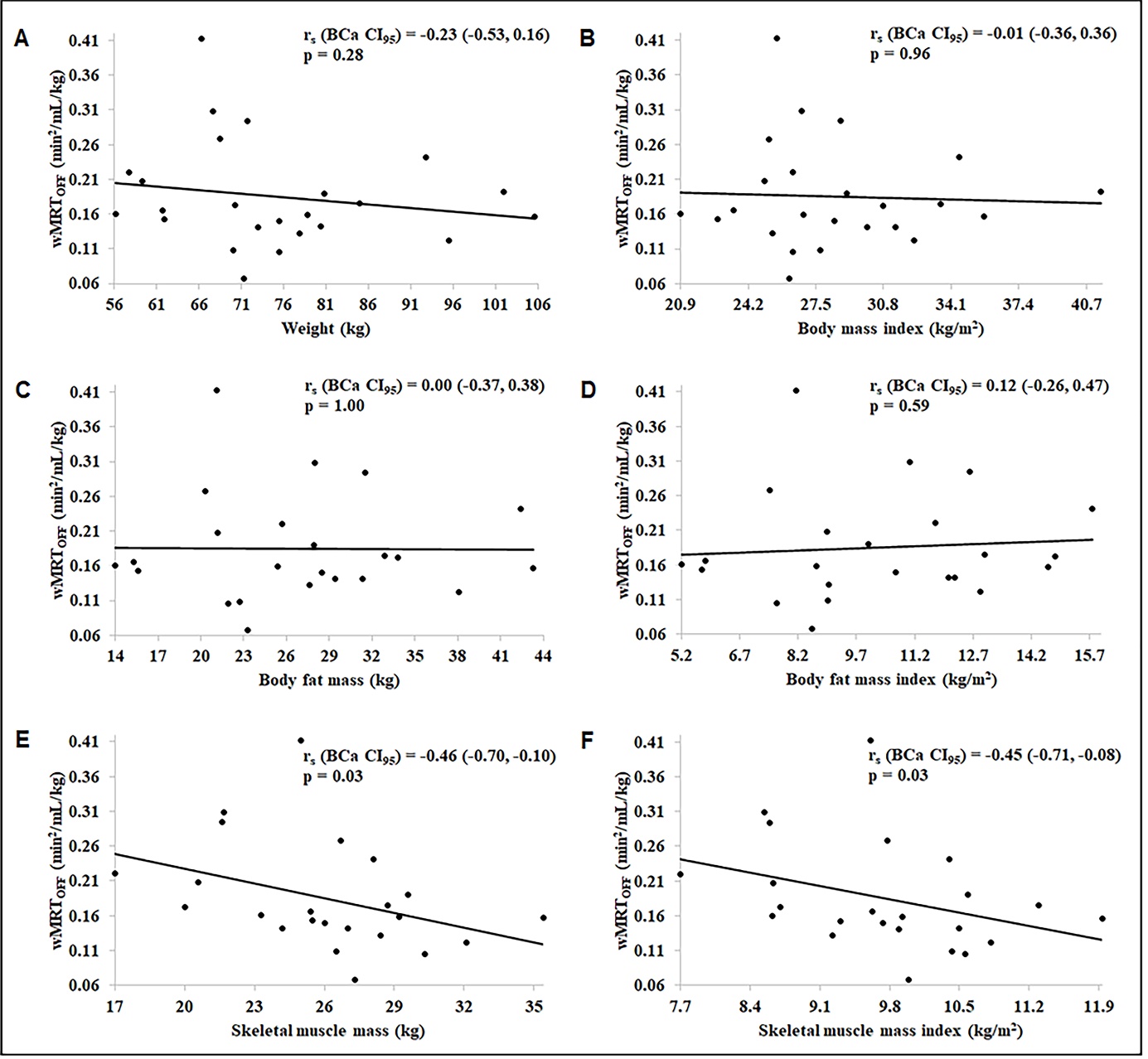
**

**S2 Fig** Relationship between the body composition and the oxygen uptake off-kinetics mean response time corrected for work rate. BCa, bias corrected accelerated; CI_95_, 95% confidence interval; kg, kilogram; kg/m^2^, kilogram per meter^2^; min^2^/mL/kg, minute square per milliliter per kilogram; r_s_, Spearman’s rank correlation coefficient; wMRT_OFF_, oxygen uptake off-kinetics mean response time corrected for work rate.

**
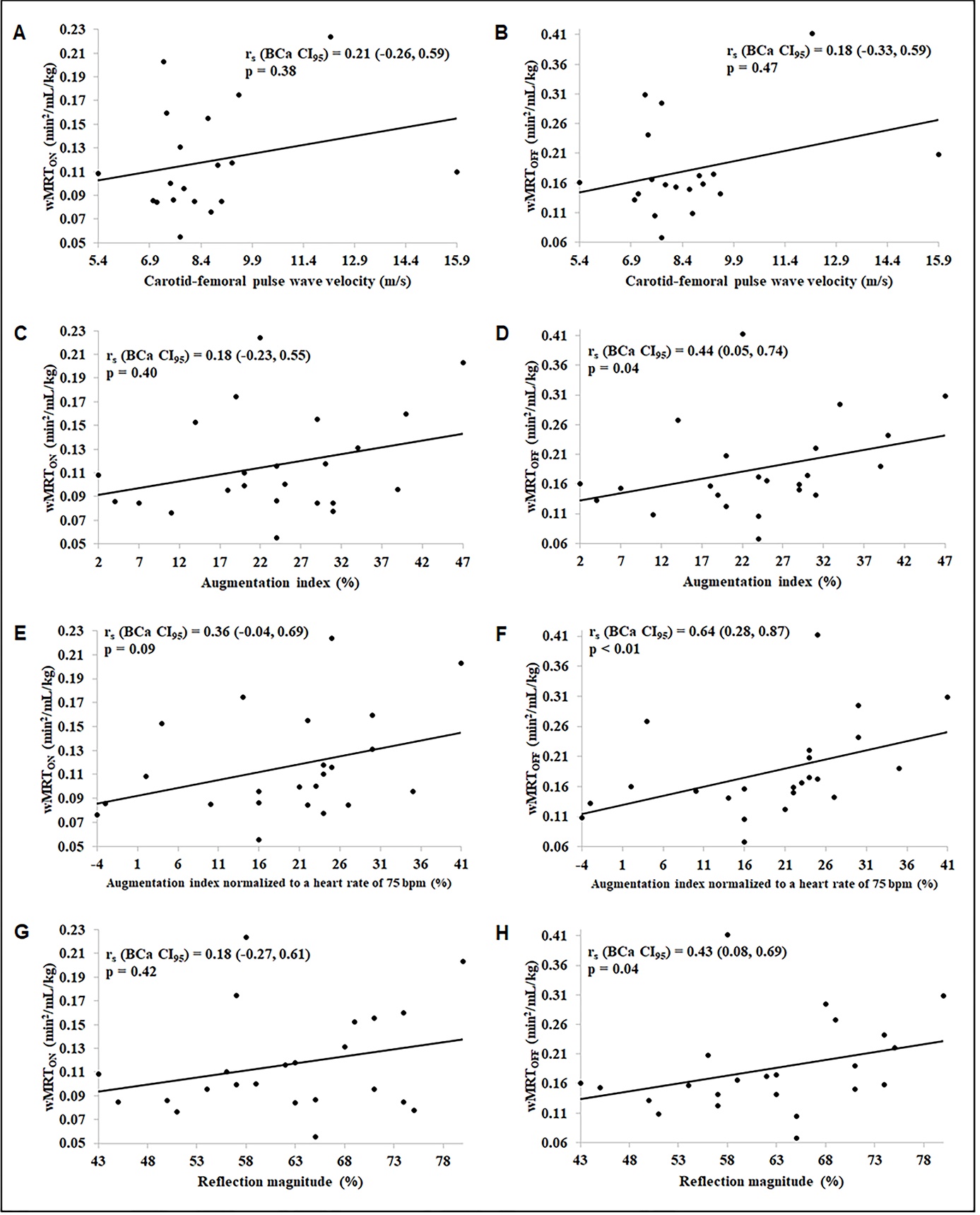
**

**S3 Fig** Relationship between the arterial compliance and the oxygen uptake kinetics. %, percentage; BCa, bias corrected accelerated; CI_95_, 95% confidence interval; m/s, meter per second; min^2^/mL/kg, minute square per milliliter per kilogram; r_s_, Spearman’s rank correlation coefficient; wMRT_OFF_, oxygen uptake off-kinetics mean response time corrected for work rate; wMRT_ON_, oxygen uptake on-kinetics mean response time corrected for work rate.

**
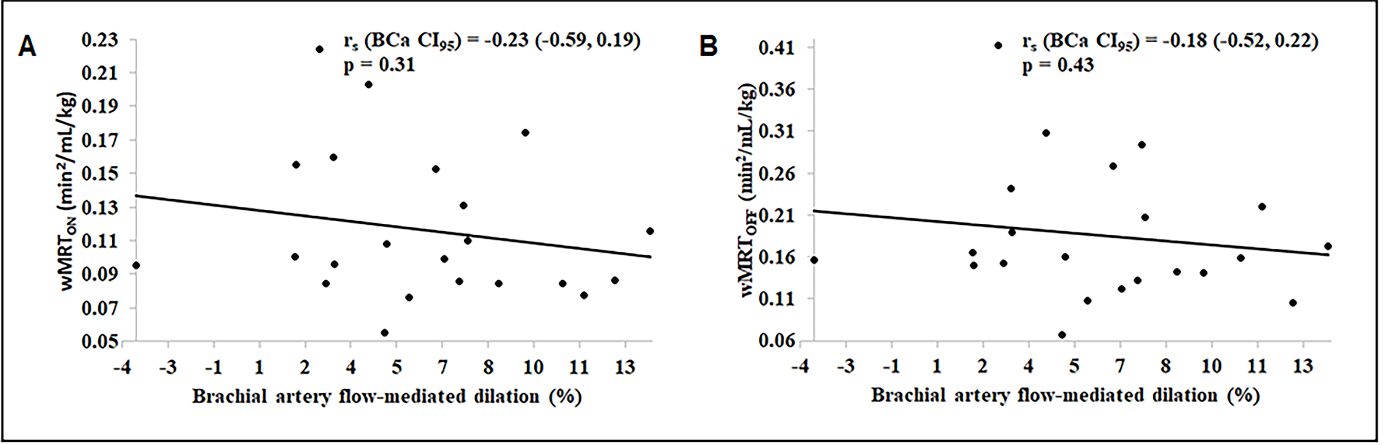
**

**S4 Fig** Relationship between the endothelial function and the oxygen uptake kinetics. %, percentage; BCa, bias corrected accelerated; CI_95_, 95% confidence interval; min^2^/mL/kg, minute square per milliliter per kilogram; r_s_, Spearman’s rank correlation coefficient; wMRT_OFF_, oxygen uptake off-kinetics mean response time corrected for work rate; wMRT_ON_, oxygen uptake on-kinetics mean response time corrected for work rate.

**
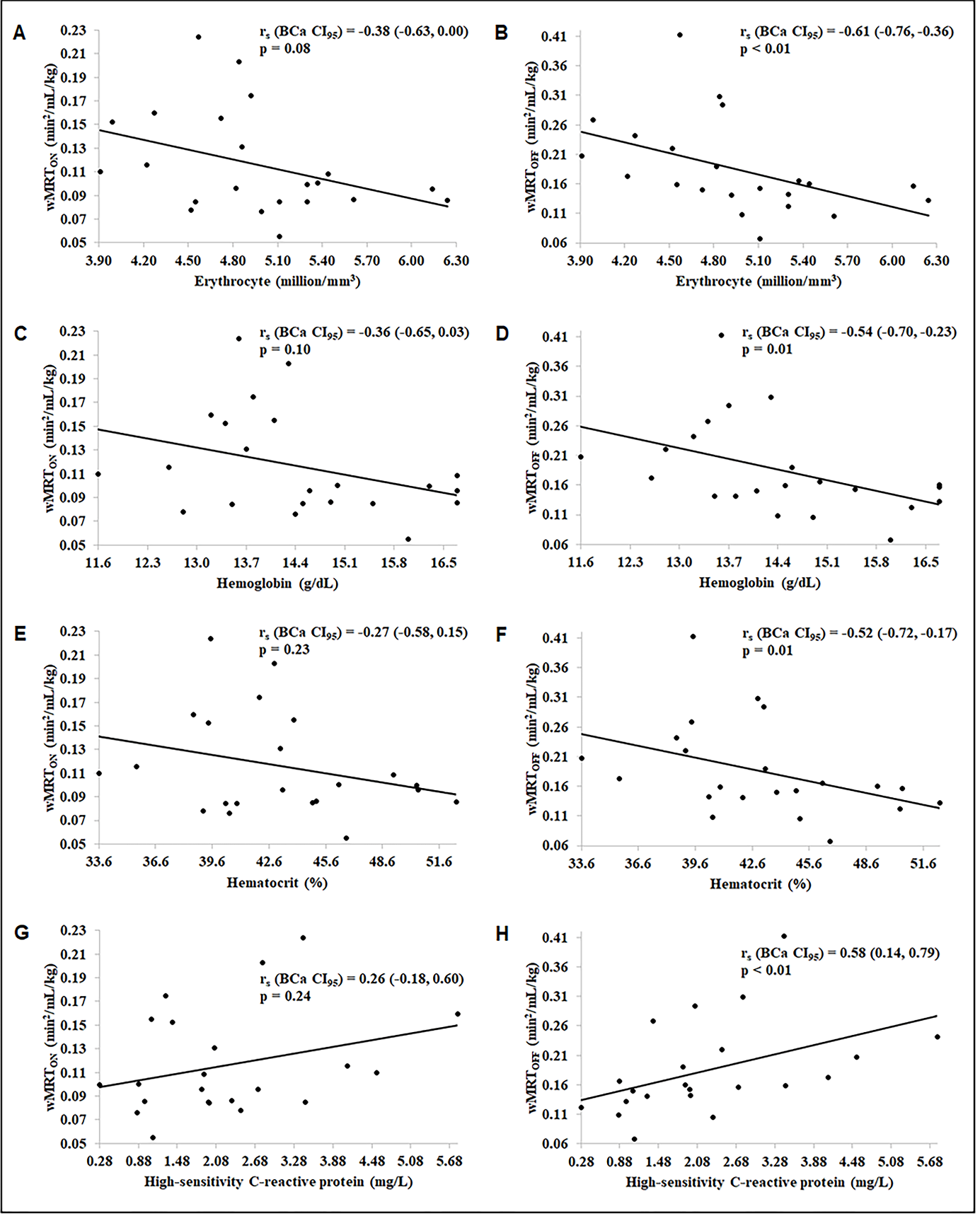
**

**S5 Fig** Relationship between the hematological and inflammatory profiles and the oxygen uptake kinetics. %, percentage; BCa, bias corrected accelerated; CI_95_, 95% confidence interval; g/dL, grams per deciliter; mg/L, milligram per liter; million/mm^3^, million per cubic millimeter; min^2^/mL/kg, minute square per milliliter per kilogram; r_s_, Spearman’s rank correlation coefficient; wMRT_OFF_, oxygen uptake off-kinetics mean response time corrected for work rate; wMRT_ON_, oxygen uptake on-kinetics mean response time corrected for work rate.

**
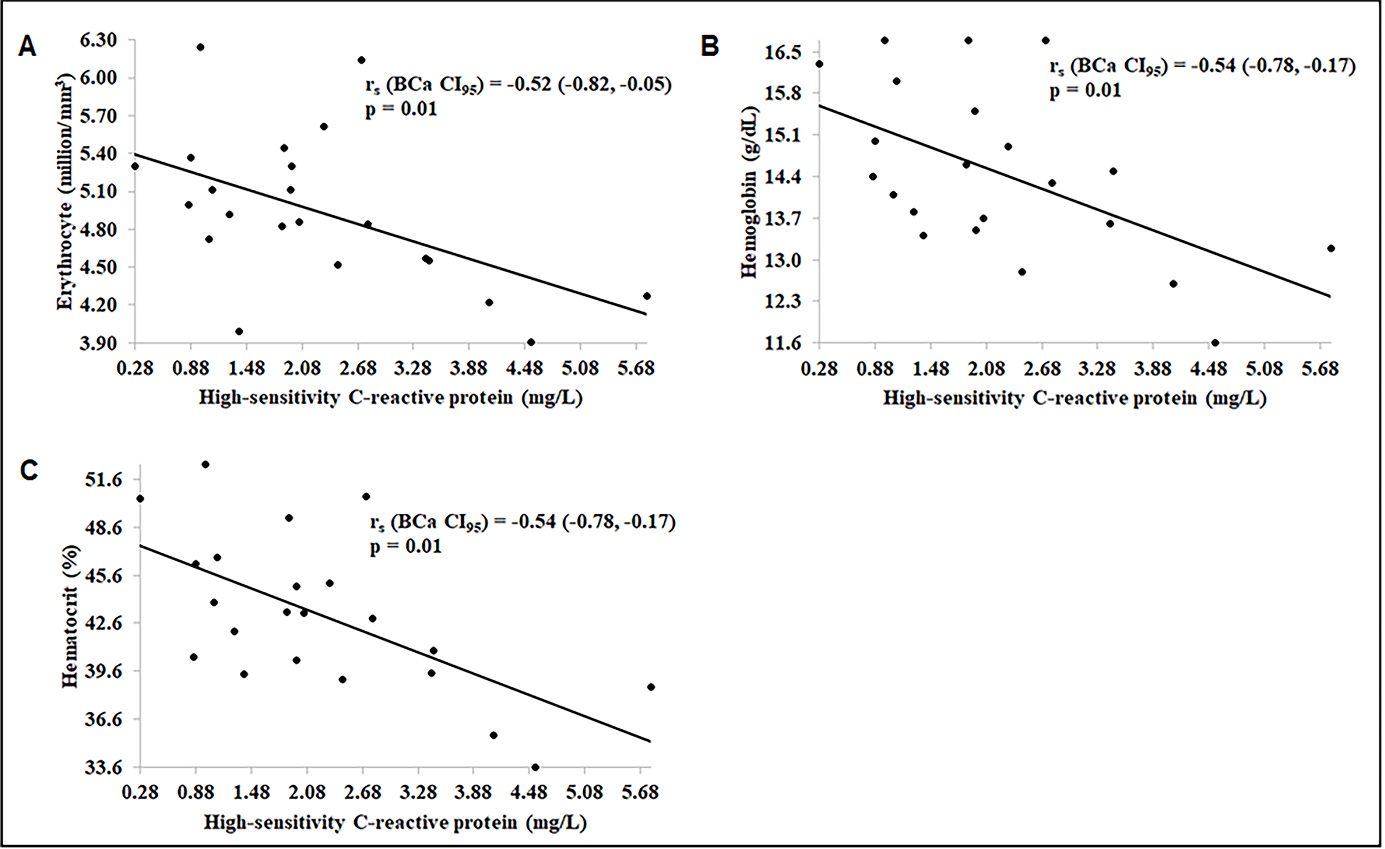
**

**S6 Fig** Relationship between the high-sensitivity C-reactive protein and hematological profile. %, percentage; BCa, bias corrected accelerated; CI_95_, 95% confidence interval; g/dL, grams per deciliter; mg/L, milligram per liter; million/mm^3^, million per cubic millimeter; r_s_, Spearman’s rank correlation coefficient.


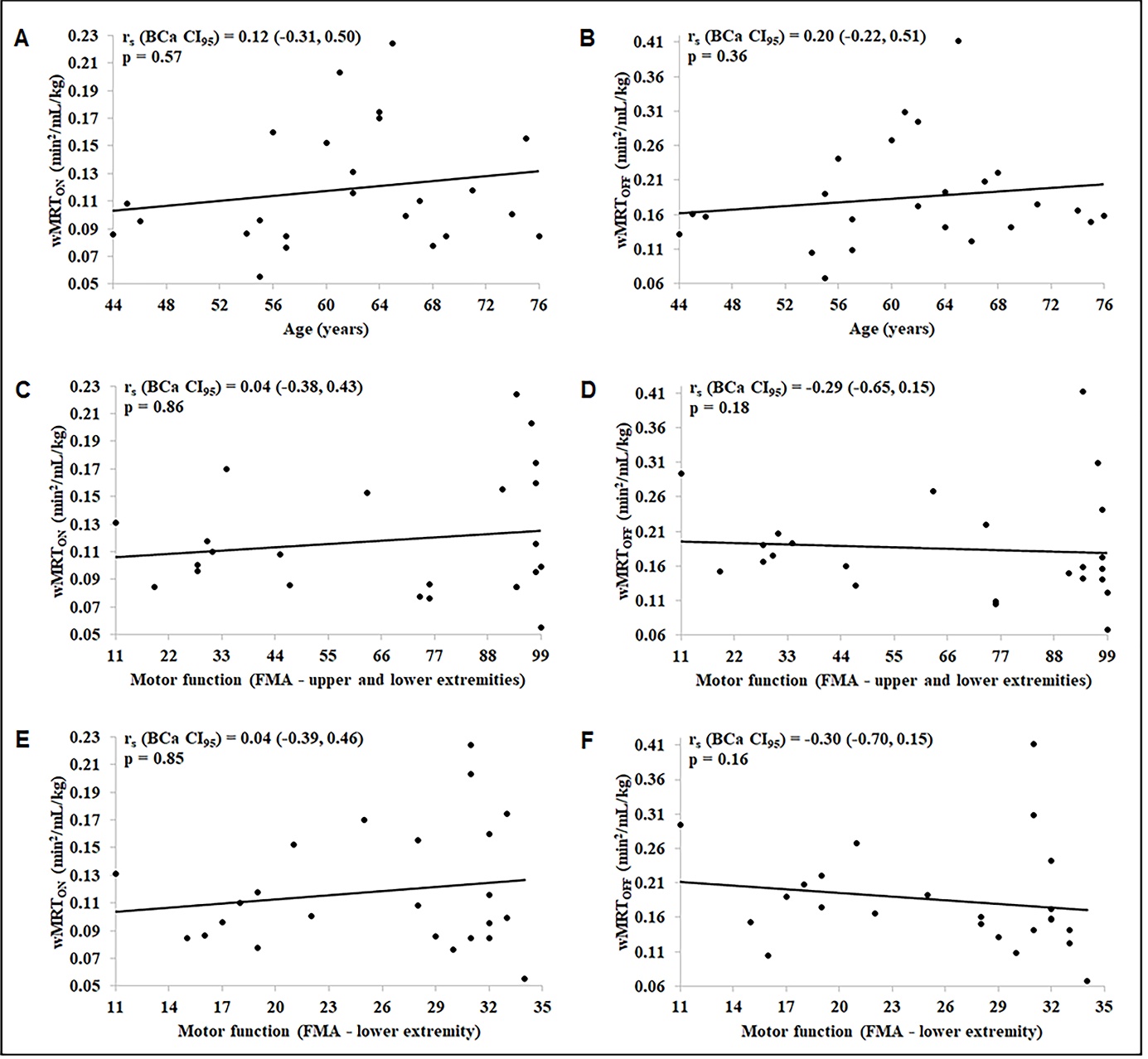


**S7 Fig** Relationship between the age, motor function and the oxygen uptake kinetics. BCa, bias corrected accelerated; CI_95_, 95% confidence interval; FMA, Fugl-Meyer Assessment of Motor Recovery after Stroke; min^2^/mL/kg, minute square per milliliter per kilogram; r_s_, Spearman’s rank correlation coefficient; wMRT_OFF_, oxygen uptake off-kinetics mean response time corrected for work rate; wMRT_ON_, oxygen uptake on-kinetics mean response time corrected for work rate.
